# Efficient Quantum Private Comparison without Sharing a Key

**DOI:** 10.3390/e25111552

**Published:** 2023-11-17

**Authors:** Jian Li, Fanting Che, Zhuo Wang, Anqi Fu

**Affiliations:** 1 School of Information Engineering, Ningxia University, Yinchuan 750021, China; lijian@bupt.edu.cn; 2 School of Cyberspace Security, Beijing University of Posts and Telecommunications, Beijing 100876, China; sirius1120@126.com; 3 School of Artificial Intelligence, Beijing University of Posts and Telecommunications, Beijing 100876, China; zhuowang@bupt.edu.cn

**Keywords:** quantum private comparison, GHZ-like states, unitary operation, decoy photon

## Abstract

Quantum private comparison (QPC) allows at least two users to compare the equality of their secret information, for which the security is based on the properties of quantum mechanics. To improve the use of quantum resources and the efficiency of private comparison, a new QPC protocol based on GHZ-like states is proposed. The protocol adopts unitary operations to encode the secret information instead of performing quantum key distribution (QKD), which can reduce the amount of computation required to perform QKD and improve the utilization of quantum resources. The decoy photon technique used to detect channel eavesdropping ensures that the protocol is resistant to external attacks. The quantum efficiency of the protocol reaches 66%. Compared with many previous QPC schemes, the proposed protocol does not need to share a key and has advantages in quantum efficiency and quantum resources.

## 1. Introduction

In the traditional field of information security, encryption technology has been the key to protecting sensitive information. However, with the rapid development of computing power, especially the development of quantum computing, securing traditional encryption methods has been substantially challenging. In this context, research on quantum cryptography, including quantum key distribution (QKD), quantum digital signature [1], quantum communication [2], and quantum private comparison (QPC), has become a research hotspot. 

QKD technology primarily relies on the fundamental principles of quantum mechanics to ensure that users generate secure and dependable keys during the communication progress [3,4]. Moreover, the goal of quantum private comparison is to enable both parties to compare their secret data without revealing any information about the data to each other or any potential eavesdropper [5,6,7]. QPC has potential applications in a variety of fields, including secure online voting, financial transactions, and data sharing between government agencies. However, the majority of QPC protocols have a common feature that the protocols need to perform QKD [8] and then encrypt and compare secret information. Using QKD to avoid possible security risks can make the protocol simpler and easier at the physical implementation level; nevertheless, this type of QPC protocol has room for improvement in quantum efficiency and utilization of quantum resources [9].

For a QPC protocol, the privacy security issue is an unavoidable research focus. A qualified QPC protocol should meet the following two conditions: (1) the security of the private information and (2) the fairness of the comparison results. Both parties need to know the final result of the comparison and ensure that the result is the same as that of the actual calculation. Therefore, it is imperative to process or encrypt the secret information, guaranteeing that the parties involved in the comparison do not have direct access to each other’s secret data and eliminating the possibility of inferring secret information from measurement results. Furthermore, the involvement of a third party in the protocol can facilitate secure and equitable private comparison by assisting the two parties in comparing their secret data and publicly publishing the comparison result. However, the integrity and trustworthiness of the third party are crucial considerations. Ensuring the credibility of the third party and the smooth progress of the protocol necessitates the implementation of necessary measures to safeguard and regulate the behavior of the third party.

Based on the above analysis, we propose a new quantum privacy comparison protocol without a shared key. With the assistance of a semi-honest third party, this protocol ensures fairness in comparing users’ secret information without the necessity for key sharing or direct user-to-user communication. This results in heightened privacy protection capabilities. Furthermore, the protocol incorporates decoy photon technology and hash functions to defend against internal and external attacks, effectively securing the performance of this protocol. Notably, this protocol boasts a higher quantum efficiency compared to the majority of previous QPC protocols.

The structure of this paper is as follows: the related work and the knowledge preparation are introduced in Section 2. The steps and description of the protocol are presented in Section 3. An example of the correctness of the protocol is shown in Section 4. The security analysis is explained in detail in Section 5. The quantum efficiency is analyzed in Section 6. Finally, a summary of this work is provided.

## 2. Preliminaries

### 2.1. Related Work

The earliest QPC protocol was inspired by quantum secret sharing protocols, and the privacy comparison problem originating from Yao’s millionaires problem. In this problem, two millionaires sought to ascertain their relative wealth without revealing specific assets. The first QPC protocol was introduced by Yang and Wen in 2009 [10]. This protocol harnessed the entanglement exchange properties of EPR states and employed unitary operations to facilitate the comparison of private information. Additionally, the involvement of a third-party participant effectively ensured the security and fairness of the protocol. In current research, QPC protocols aim to compare the equality or the relative sizes of private information. The research predominantly focuses on using different quantum states and various encoding methods (whether to distribute keys) to implement comparison protocols.

In 2020, Lang [11] introduced a QPC protocol that leveraged quantum gates, simplifying the process by eliminating the classical computation in a QPC protocol. In the same year, Wu et al. [12] proposed a protocol that does not require the involvement of a third party, and Ji et al. [13] designed several QPC protocols with various quantum states, using dense coding instead of key distribution methods. In 2021, Huang et al. [14] introduced a semi-honest third party to assist in comparison. Lang [15] also proposed a QPC protocol using a single Bell state rather than multiple Bell states as the quantum resource. Chen et al. designed a QPC protocol that does not require the use of quantum entangled states as quantum resources [16]. In 2022, Fan et al. [17] utilized an eight-qubit entangled state for private data comparison through quantum key distribution and joint computation. In 2023, Liu [18] employed high-dimensional GHZ-like states as quantum resources, and Zhang et al. [19] introduced a QPC protocol based on homomorphic encryption, allowing multiple participants to engage in simultaneous comparisons.

### 2.2. Theoretical Basis of GHZ-like States

GHZ-like states are a class of quantum states that have certain similarities to GHZ states, such as multi-partite entanglement, where the entanglement between these qubits of GHZ-like states is multipartite rather than just bipartite. Moreover, the GHZ-like state is not limited to specific forms of GHZ states.

The three-particle GHZ-like states used in the proposed QPC protocol are transformed from ordinary GHZ states. An n-particle GHZ state is a kind of quantum entanglement, which can be described as

(1) $$|GHZ\rangle =\frac{1}{\sqrt{2}}\left(|q_{1},q_{2},\cdots ,q_{n}\rangle +{\left(-1\right)}^{\Delta }|\bar{q_{1},q_{2},\cdots ,q_{n}}\right)$$

where $q_{1}=0$, $q_{2},q_{3},\cdots ,q_{n}\in \left\{0,1\right\}$ and $\Delta \in \left\{0,1\right\}$.

According to Equation (1), an n-particle GHZ-like state [20] transformed from the GHZ state can be written as

(2) $${|GHZ\rangle }^{\prime }=\frac{1}{\sqrt{2^{n-1}}}\overset{2^{n-1}}{\underset{k=1}{\sum }}{\left[{\left(-1\right)}^{\delta }|z_{1},z_{2},\cdots ,z_{n}\rangle \right]}_{num\left(1\right)}$$

where $\delta =\left(\underset{j|z_{j}=1}{\sum }q_{j}\right)\left(mod2\right)$, $z_{j}\in \left\{0,1\right\}$, and $num\left(1\right)=\left\{\begin{array}{c} \mathrm{even},\;\mathrm{if}\;\Delta =0\\ \mathrm{odd},\;\mathrm{if}\;\Delta =1 \end{array}\right.$. When the parameter *num*(1) is an even value, the number of 1 in each vector $|z_{1},z_{2},\cdots ,z_{n}\rangle$ is even. When the parameter *num*(1) is an odd value, the number of 1 in each vector $|z_{1},z_{2},\cdots ,z_{n}\rangle$ is odd.

According to Equation (1), an n-particle GHZ state can exit $2^{n}$ different states. It should be noted that the state is a Bell state when n takes the value of 2. The Bell state is the simplest quantum entangled state of a two-qubit system, consisting of four specific maximum entangled quantum states, which can be expressed as Equations (3) and (4). For a three-particle GHZ state, there are eight possible states and eight corresponding GHZ-like states. The one used in the proposed protocol is ${|GHZ\rangle }_{1}$, in which the Δ has a value of 0 and $q_{2}=q_{3}=0$. ${|GHZ\rangle }_{1}$ is a GHZ state, which is shown as Equation (5).

(3) $$|\Phi^{+}\rangle =\frac{1}{\sqrt{2}}\left[|00\rangle +|11\rangle \right],|\Phi^{-}\rangle =\frac{1}{\sqrt{2}}\left[|00\rangle -|11\rangle \right]$$

(4) $$|\Psi^{+}\rangle =\frac{1}{\sqrt{2}}\left[|01\rangle +|10\rangle \right],|\Psi^{-}=\frac{1}{\sqrt{2}}\left[|01\rangle -|10\rangle \right]$$

(5) $${|GHZ\rangle }_{1}=\frac{1}{\sqrt{2}}\left(|000\rangle +|111\rangle \right)$$

According to Equations (1) and (2), the GHZ-like state ${{|GHZ\rangle }_{1}}^{\prime }$ used in the proposed protocol can be described as

(6) $$\begin{array}{cc} {{|GHZ\rangle }_{1}}^{\prime }=\frac{1}{\sqrt{2^{2}}} & \overset{2^{2}}{\underset{k=1}{\sum }}{\left[{\left(-1\right)}^{\delta }|z_{1},z_{2},\cdots ,z_{n}\rangle \right]}_{even}\\ & =\frac{1}{2}\left[{\left(-1\right)}^{0}|000\rangle +{\left(-1\right)}^{0⊕0}|011\rangle +{\left(-1\right)}^{0⊕0}|101\rangle +{\left(-1\right)}^{0⊕0}|110\rangle \right]\\ & \;\;=\frac{1}{2}\left[|000\rangle +|011\rangle +|101\rangle +|110\rangle \right] \end{array}$$

The ${{|GHZ\rangle }_{1}}^{\prime }$ also can be written as

(7) $${{|GHZ\rangle }_{1}}^{\prime }=\frac{1}{2}\left[|0\rangle \left(|00\rangle +|11\rangle \right)+|1\rangle \left(|01\rangle +|10\rangle \right)\right]=\frac{1}{\sqrt{2}}\left[|0\rangle |\Phi^{+}\rangle +|1\rangle |\Psi^{+}\rangle \right]$$

According to Equation (7), when the state of the first particle of ${{|GHZ\rangle }_{1}}^{\prime }$ is $|0\rangle$, the state of the other two particles of ${{|GHZ\rangle }_{1}}^{\prime }$ corresponds to the Bell state $|\Phi^{+}\rangle$. When the state of the first particle of ${{|GHZ\rangle }_{1}}^{\prime }$ is $|1\rangle$, the state of the rest particles of ${{|GHZ\rangle }_{1}}^{\prime }$ corresponds to the Bell state $|\Psi^{+}\rangle$. These properties of ${{|GHZ\rangle }_{1}}^{\prime }$ are used in our proposed protocol.

### 2.3. Unitary Operations

The unitary operations used in the proposed protocol are Pauli gates. Pauli gates (X, Y, Z) are three quantum gates, which operate on a single qubit. The Pauli-X gate flips the state of a qubit from $|0\rangle$ to $|1\rangle$ and vice versa. The Pauli-Y gate changes the state of a qubit from $|0\rangle$ to $-|1\rangle$ and $|1\rangle$ to $|0\rangle$. The Pauli-Z gate changes the state of a qubit from $|1\rangle$ to $|0\rangle$ and $|1\rangle$ to $-|1\rangle$. Shown in Table 1, the outcomes of the state of a qubit, which passes through a Pauli gate or Identity gate, are listed.

## 3. QPC Protocol Description

The protocol participants are introduced as follows:

**TP:** TP is a semi-honesty third party that can help compare the equality of the secret information. TP needs to honestly execute the steps specified in the protocol but allows it to attempt to obtain secret information through some illegal means.

**Alice (Bob):** Alice (Bob) is a quantum user with complete quantum capabilities who can achieve the preparation and complete measurement of quantum states.

**Figure 1:**
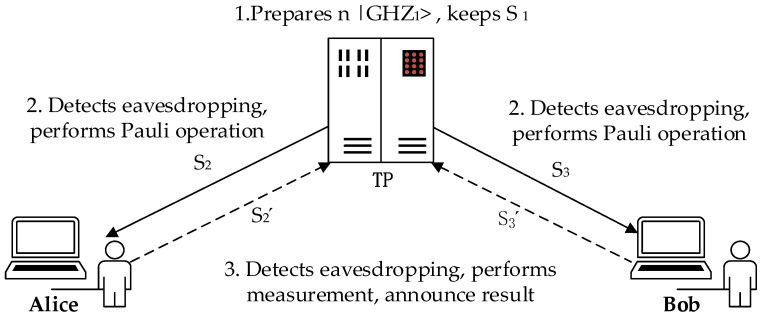
The model of the proposed protocol.

Assume the length of the secret information that Alice and Bob need to compare is *L*. The secret information of Alice can be expressed as $X=\left\{x_{1},x_{2},\cdots ,x_{L}\right\}$, and the secret information of Bob can be expressed as $Y=\left\{y_{1},y_{2},\cdots ,y_{L}\right\}$, where $x_{i}$ and $y_{i}$ consist of the classical bits 0 and 1, and *i* represents the *i*-th particle of the particle sequence *X* or *Y*. Moreover, the protocol is described in detail as follows (also shown in Figure 1).

**Step 1:** TP prepares *n* ${{|GHZ\rangle }_{1}}^{\prime }$ states and divides them into three sequences $S_{1}^{\;}$, $S_{2}^{\;}$, and $S_{3}^{\;}$, where $S_{i}^{\;}$ includes all of the *i*-th particles of each state.

**Step 2:** TP generates 2*m* decoy photons. Each photon is prepared randomly from four states of single particles |0〉, |1〉, |+〉, and |−〉. Thereafter, TP chooses m decoy photons and randomly inserts them into $S_{2}^{\;}$ and then randomly inserts the remaining m decoy photons into $S_{3}^{\;}$. The position of insertion is random. After completing the insertion, the total number of particles of the particle string is *n + m*. TP records the location and state of these decoy photons. The value of m can be an arbitrary number, but it should be large enough to improve the performance of eavesdropping detection.

**Step 3:** TP keeps the sequence $S_{1}^{\;}$ for his/her own and sends $S_{2}^{\;}$ and $S_{3}^{\;}$ to Alice and Bob, respectively.

**Step 4:** Upon receiving the sequences sent by TP, both Alice and Bob immediately conduct eavesdropping detection. Meanwhile, TP announces the location and basis of each decoy photon. Then, Alice and Bob perform the corresponding measurement on these decoy photons, send the measurement results to TP, and discard the decoy photons in $S_{2}^{\;}$ and $S_{3}^{\;}$. Thereafter, TP determines whether the eavesdropper is on the transmission channel based on the received measurement results. If the error ratio of the measurement results exceeds a predefined threshold, it indicates the presence of eavesdroppers on the communication channel and the protocol needs to be terminated immediately.

**Step 5:** Alice and Bob perform a shared hash function *H* on their binary secret information to obtain a binary sequence of the specified length. The hash function *H* is described as Equation (8).

(8) $$H:{\left(0,\;1\right)}^{L}\to {\left(0,\;1\right)}^{K}$$

where *L* denotes the initial length of the binary sequence and *K* denotes the length of the binary sequence after performing the shared hash function *H*.

(9) $$H:X=\left\{x_{1},x_{2},\cdots ,x_{L}\right\}\to X^{\prime }=\left\{x_{1},x_{2},\cdots ,x_{K}\right\}$$

(10) $$H:Y=\left\{y_{1},y_{2},\cdots ,y_{L}\right\}\to X^{\prime }=\left\{y_{1},y_{2},\cdots ,y_{K}\right\}$$

where *K* gets the value of 2*n* in the protocol.

Thereafter, Alice and Bob divide $X^{\prime }$ and $Y^{\prime }$ into 2*n* groups, respectively.

(11) $$X^{\prime }=\left\{\left(x_{1}{}^{\prime },x_{2}{}^{\prime }\right),\left(x_{3}{}^{\prime },x_{4}{}^{\prime }\right),\cdots ,\left(x_{2n-1}{}^{\prime },x_{2n}{}^{\prime }\right)\right\}=\left\{m_{1}{}^{A},m_{2}{}^{A},\cdots ,m_{n}{}^{A}\right\}$$

(12) $$Y^{\prime }=\left\{\left(y_{1}{}^{\prime },y_{2}{}^{\prime }\right),\left(y_{3}{}^{\prime },y_{4}{}^{\prime }\right),\cdots ,\left(y_{2n-1}{}^{\prime },y_{2n}{}^{\prime }\right)\right\}=\left\{m_{1}{}^{A},m_{2}{}^{A},\cdots ,m_{n}{}^{A}\right\}$$

where $m_{i}\in \left\{00,\;01,\;10,\;11\right\}$.

**Step 6:** Alice and Bob perform a corresponding operation, which is shown in Table 2, on each particle in $S_{2}^{\;}$ and $S_{3}^{\;}$. After all the particles have been manipulated, Alice and Bob prepare *m* decoy photons each, insert them into $S_{2}^{\prime }\;$ and $S_{3}^{\prime }\;$ composed of the operation results, and send the sequences $S_{2}^{\prime }$ and $S_{3}^{\prime }\;$ back to TP.

**Step 7:** After receiving $S_{2}^{\prime }$ and $S_{3}^{\prime }$, TP carries out eavesdropping detection immediately to ensure that there is no eavesdropping. TP discards the decoy photons after eavesdropping detection.

**Step 8:** TP combines both sequences to form the $Q_{i}=\left(q_{2}^{i},q_{3}^{i}\right)$, in which the $q_{2}^{i}$ is the *i*-th particle of $S_{2}^{\prime }$ and the $q_{3}^{i}$ is the *i*-th particle of $S_{3}^{\prime }$. Then, TP measures the pairs $Q_{i}$ with Bell basis and measures each particle of the sequence $S_{1}^{\;}$ with $|0,1\rangle$ basis.

**Step 9:** TP judges the equality of the secret information of Alice and Bob based on the measurement results in Step 8. If the measurement results of Bell basis and $|0,1\rangle$ basis are $|\Phi^{+}\rangle$ and $|0\rangle$ or $|\Psi^{+}\rangle$ and $|1\rangle$, the secret information being compared is equal. Otherwise, if the measurement result occurs in another situation, the secret information being compared is different. The equal results are shown in Table 3, and the summary of all measurement results is shown in Table 4.

## 4. Correctness

Suppose the quantum users Alice and Bob want to compare the secret information X=Y=101100011001, which would turn into $X^{\prime }$ and $Y^{\prime }$ after performing the shared hash function *H* (Shown as Equation (8)). The purpose of performing the hash operation is to convert the comparative secret information to a specific length and to perform the first encryption processing. For convenience of presentation, we assume that $X^{\prime }=Y^{\prime }=10110001$.

TP prepares four three-particle GHZ-like states and divides them into three sequences $S_{1}^{\;}=s_{1}^{1},s_{1}^{2},s_{1}^{3},s_{1}^{4}$, $S_{2}^{\;}=s_{2}^{1},s_{2}^{2},s_{2}^{3},s_{2}^{4}$ and $S_{3}^{\;}=s_{3}^{1},s_{3}^{2},s_{3}^{3},s_{3}^{4}$. Then, TP inserts decoy photons into $S_{2}^{\;}$ and $S_{3}^{\;}$. Thereafter, TP sends $S_{2}^{\;}$ and $S_{3}^{\;}$ to Alice and Bob, respectively. After receiving the sequences, Alice and Bob perform eavesdropping detection immediately and carry out the operations $\left\{Y,Z,I,X\right\}$ based on the result of secret information performed by the hash function H. Then, Alice and Bob prepare decoy photons each and send $S_{2}^{\prime }$ and $S_{3}^{\prime }$ back to TP.

TP performs eavesdropping detection and carries out the Bell basis and $|0,1\rangle$ basis measurement. The measurement results are $|\Phi^{+}\rangle$ and $|0\rangle$, $|\Psi^{+}\rangle$ and $|1\rangle$. The result means that the secret information being compared is equal. The comparison process is shown in Table 5, and an example of unequal secret information is shown in Table 6. The decoy photons are not displayed in the comparison process shown in Table 5 and Table 6. The decoy photons are randomly located in the quantum sentences and discarded after eavesdropping detection.

## 5. Security Analyses

### 5.1. External Attacks

Assume the external attacker is Eve. The attack methods that Eve can use to steal the secret information of Alice or Bob or both Alice and Bob are intercept-resend attack, measure-resend attack, and entanglement attack. The following is a detailed analysis of these three attacks.

#### 5.1.1. Intercept-Resend Attack

The external attacker Eve may carry out an intercept-resend attack by first intercepting the particle sequences sent by TP to Alice and Bob and storing them. Then, Eve prepares the same and specified amount of single particles and sends them to Alice and Bob. After Alice and Bob perform their operations, Eve again intercepts the quantum particle sequences sent by Alice and Bob to TP and makes measurements to obtain secret information about Alice and Bob, while returning the previously stored quantum particle sequences to TP.

However, Eve’s attack will inevitably introduce errors because Alice, Bob, and TP will perform eavesdropping detection as soon as they receive the particle sequence. The receivers will require the sender to disclose the location and measurement basis of the bait particles, and Eve cannot know the specific state of these particles. When the receiver selects the measurement basis announced by the sender to measure the single particle sent by Eve, there is a 50% probability of obtaining an incorrect result. Eve does not prepare a single particle with the same state as the particle sequences, for example, the original decoy photon has a state of $|1\rangle$, but Eve prepares a single particle with a state of $|0\rangle$. The probability of Eve preparing particles in the wrong state is 1/2. The probability of Eve successfully deceiving the detection is ${\left(1/2\right)}^{n}$, where *n* is the number of decoy photons measured in the eavesdropping detection. When the value of *n* is large enough, the probability of Eve being discovered is infinitely close to 1. Therefore, the intercept-resend attack is invalid for the method of the present invention.

#### 5.1.2. Measure-Resend Attack

The external attacker Eve can execute the measure-resend attack by first intercepting the particle sequences sent by TP to Alice and Bob and performing $|0,1\rangle$ basis measurement. Then, based on the measurement results, new quantum particles are prepared and sent to Alice and Bob. After Alice and Bob complete their operations, Eve intercepts the quantum particle sequence sent by Alice and Bob to TP again and conducts measurements to try to obtain the secret information encoded in the quantum particle sequences. At the same time, a new quantum particle sequence is prepared based on the measurement results and transmitted back to TP.

Nonetheless, Eve’s attack will unavoidably result in errors because the decoy photons prepared by Alice, Bob, and TP have four states, $|0\rangle$, $|1\rangle$, $|+\rangle$, and $|-\rangle$. The state $|0\rangle$ and $|1\rangle$ can be measured using the $|0,1\rangle$ basis. The state $|+\rangle$ and $|-\rangle$ can be measured using the X basis. Moreover, Eve cannot know the position of these decoy photons in the state $|+\rangle$ and $|-\rangle$, so it is impossible to prepare corresponding quantum particles and send them to the receivers. Eve may choose the $|0,1\rangle$ basis to measure these quantum particles to get much more secret information. When the receivers conduct eavesdropping detection, the senders announce the position and measurement basis of the decoy photons, and the receivers select the corresponding measurement basis for measurement. If the measurement basis is $|0,1\rangle$ basis, no errors will be found; if the measurement basis is X, there is a 50% probability of obtaining incorrect results (shown in Table 7). Therefore, the measure-resend attack is invalid for the method of the present invention.

#### 5.1.3. Entanglement Attack

The external attacker Eve can perform the entanglement attack by first entangling her auxiliary quantum particles $|\varepsilon \rangle$ with the target particle; then, Eve measures her auxiliary particles to obtain useful information. The proposed protocol uses a bidirectional quantum channel for quantum communication, so Eve’s entanglement measurement attack can be modeled as two unitary operations $U_{E}$ and $U_{F}$. $U_{E}$ is performed on the channel TP to Alice and Bob, while $U_{F}$ is performed on the channel Alice and Bob to TP. When the proposed protocol performs eavesdropping detection, only decoy particles are measured, and if Eve can deceive the detection in this stage, the attack can be undetected. Therefore, the following analysis demonstrates that the decoy particle technology applied in the present invention can resist Eve’s entanglement attack.

When Eve uses the unitary operation $U_{E}$ to entangle with the particle that stays in the state $|0\rangle$ and $|1\rangle$, the state of $|0\rangle$ or $|1\rangle$ has been changed. Its state can be reformulated as:

(13) $$U_{E}{|0,\varepsilon \rangle }_{TE}=\lambda_{00}|0\rangle |\epsilon_{00}\rangle +\lambda_{01}|1\rangle |\epsilon_{01}\rangle$$

(14) $$U_{E}{|1,\varepsilon \rangle }_{TE}=\lambda_{10}|0\rangle |\epsilon_{10}\rangle +\lambda_{11}|1\rangle |\epsilon_{11}\rangle$$

where T and E represent the decoy particles of users and the auxiliary particles of Eve. $|\epsilon_{00}\rangle$, $|\epsilon_{01}\rangle$, $|\epsilon_{10}\rangle$, and $|\epsilon_{11}\rangle$ represent the pure states selected by Eve in the unitary operation $U_{E}$. $\lambda_{00}$, $\lambda_{01}$, $\lambda_{10}$, and $\lambda_{11}$ must meet the conditions: ${\left\|\lambda_{00}\right\|}^{2}+{\left\|\lambda_{01}\right\|}^{2}=1$, ${\left\|\lambda_{10}\right\|}^{2}+{\left\|\lambda_{11}\right\|}^{2}=1$.

The decoy particles $|+\rangle$ and $|-\rangle$ can be expressed as

(15) $$\begin{array}{c} |+\rangle =\frac{1}{\sqrt{2}}\left(|0\rangle +|1\rangle \right),|-\rangle =\frac{1}{\sqrt{2}}\left(|0\rangle -|1\rangle \right) \end{array}$$

When Eve uses the auxiliary particle $|\varepsilon \rangle$ to entangle with $|+\rangle$ or $|-\rangle$ in the operation $U_{E}$, the state of $|+\rangle$ or $|-\rangle$ has been changed. And its state can be reformulated as

(16) $$U_{E}{|+,\varepsilon \rangle }_{TE}=\frac{1}{\sqrt{2}}\left(\lambda_{00}|0\rangle |\epsilon_{00}\rangle +\lambda_{01}|1\rangle |\epsilon_{01}\rangle +\lambda_{10}|0\rangle |\epsilon_{10}\rangle +\lambda_{11}|1\rangle |\epsilon_{11}\rangle \right)\;=\frac{1}{2}|+\rangle \left(\lambda_{00}|\epsilon_{00}\rangle +\lambda_{01}|\epsilon_{01}\rangle +\lambda_{10}|\epsilon_{10}\rangle +\lambda_{11}|\epsilon_{11}\rangle \right)\;\begin{array}{c} +\frac{1}{2}|-\rangle \left(\lambda_{00}|\epsilon_{00}\rangle -\lambda_{01}|\epsilon_{01}\rangle +\lambda_{10}|\epsilon_{10}\rangle -\lambda_{11}|\epsilon_{11}\rangle \right) \end{array}$$

(17) $$U_{E}{|-,\varepsilon \rangle }_{TE}=\frac{1}{\sqrt{2}}\left(\lambda_{00}|0\rangle |\epsilon_{00}\rangle +\lambda_{01}|1\rangle |\epsilon_{01}\rangle -\lambda_{10}|0\rangle |\epsilon_{10}\rangle -\lambda_{11}|1\rangle |\epsilon_{11}\rangle \right)\;=\frac{1}{2}|+\rangle \left(\lambda_{00}|\epsilon_{00}\rangle +\lambda_{01}|\epsilon_{01}\rangle -\lambda_{10}|\epsilon_{10}\rangle -\lambda_{11}|\epsilon_{11}\rangle \right)\;+\frac{1}{2}|-\rangle \left(\lambda_{00}|\epsilon_{00}\rangle -\lambda_{01}|\epsilon_{01}\rangle -\lambda_{10}|\epsilon_{10}\rangle +\lambda_{11}|\epsilon_{11}\rangle \right)$$

In the above equation, some conditions must be satisfied to avoid Eve introducing errors when the users perform eavesdropping detection.

(18) $$\begin{array}{c} \lambda_{01}=\lambda_{10}=0,\lambda_{00}|\epsilon_{00}\rangle =\lambda_{11}|\epsilon_{11}\rangle \end{array}$$

In the proposed protocol, the entanglement attack of Eve mainly acts on the second and third particles of the GHZ-like states used for transmitting information. The GHZ-like state ${{|GHZ\rangle }_{1}}^{\prime }$ used in the proposed protocol can be written as follows:

(19) $$\begin{array}{c} {{|GHZ\rangle }_{1}}^{\prime }=\frac{1}{2}\left[|0\rangle \left(|00\rangle +|11\rangle \right)+|1\rangle \left(|01\rangle +|10\rangle \right)\right]=\frac{1}{\sqrt{2}}\left[|0\rangle |\Phi^{+}\rangle +|1\rangle |\Psi^{-}\rangle \right] \end{array}$$

The second and third particles of the GHZ-like state can be considered as a Bell state together. Without losing generality, the following Bell state expressions are used for analysis.

(20) $$\begin{array}{c} |\Gamma^{\pm }\rangle =\frac{1}{\sqrt{2}}\left[|0q\rangle \pm |1\bar{q}\rangle \right] \end{array}$$

After Eve performs the unitary operation $U_{E}^{A}$ and $U_{E}^{B}$ on the two particles, respectively, the state of the two particles is changed.

(21) $$U_{E}^{A}⊗U_{E}^{B}|0q,\varepsilon_{A}\varepsilon_{B}\rangle =U_{E}^{A}|0\rangle |\varepsilon_{A}\rangle ⊗U_{E}^{B}|q\rangle |\varepsilon_{B}\rangle =\lambda_{00}|0\rangle |\epsilon_{00}\rangle ⊗\lambda_{qq}|1\rangle |\epsilon_{qq}\rangle \;=\lambda_{00}\lambda_{qq}|0q\rangle |\epsilon_{00}\rangle |\epsilon_{qq}\rangle \;$$

(22) $$U_{E}^{A}⊗U_{E}^{B}|1\bar{q},\varepsilon_{A}\varepsilon_{B}\rangle =U_{E}^{A}|1\rangle |\varepsilon_{A}\rangle ⊗U_{E}^{B}|\bar{q}\rangle |\varepsilon_{B}\rangle =\lambda_{11}|1\rangle |\epsilon_{11}\rangle ⊗\lambda_{\bar{q}\bar{q}}|\bar{q}\rangle |\epsilon_{\bar{q}\bar{q}}\rangle \;=\lambda_{11}\lambda_{\bar{q}\bar{q}}|1\bar{q}\rangle |\epsilon_{11}\rangle |\epsilon_{\bar{q}\bar{q}}\rangle =\lambda_{00}\lambda_{qq}|1\bar{q}\rangle |\epsilon_{00}\rangle |\epsilon_{qq}\rangle$$

where Equation (22) can be simplified, combined with Equation (18). The result is given as follows.

(23) $$U_{E}^{A}⊗U_{E}^{B}|\psi^{\pm },\varepsilon_{A}\varepsilon_{B}\rangle =\frac{1}{\sqrt{2}}U_{E}^{A}⊗U_{E}^{B}\left(|0q,\varepsilon_{A}\varepsilon_{B}\rangle \pm |1\bar{q},\varepsilon_{A}\varepsilon_{B}\rangle \right)\;=\frac{1}{\sqrt{2}}\left(\lambda_{00}\lambda_{qq}|0q\rangle |\epsilon_{00}\rangle |\epsilon_{qq}\rangle \pm \lambda_{00}\lambda_{qq}|1\bar{q}\rangle |\epsilon_{00}\rangle |\epsilon_{qq}\rangle \right)\;\begin{array}{c} =\lambda_{00}\lambda_{qq}|\psi^{\pm }\rangle |\epsilon_{00}\rangle |\epsilon_{qq}\rangle \end{array}$$

According to Equation (14), the tensor product of the attacker Eve’s auxiliary particle and its target particle can be expressed as a simple product of the two, so Eve’s auxiliary particle and target particle are independent of each other. Overall, if Eve does not want to introduce errors in eavesdropping detection in the present invention, Eve’s auxiliary particles and target particles are independent of each other, which means that there is no entanglement. Eve is unable to obtain information about the target particle by measuring her auxiliary particles. Hence, the entanglement attacks are ineffective against our protocol.

Above all, this protocol is impervious to external attacks. The protocol uses the decoy photon technique, which also has been proven to be unconditional security [21,22], to ensure the security of the quantum communication channel and counter most external attacks.

### 5.2. Participant Attacks

Apart from external attacks, a QPC protocol may also be attacked by internal participants. In the following, two attacks from the participants are analyzed in detail.

#### 5.2.1. The Attack from Alice or Bob

Without loss of generality, assume that Bob is a malicious user aiming to acquire Alice’s sensitive information. As mentioned in our protocol, $S_{2}^{\prime }$ is computed from Alice’s secret. Since there is no direct communication between Alice and Bob, to get Alice’s operations on each particle of $S_{2}^{\prime }$, Bob needs to intercept the sequence $S_{2}^{\;}$ sent by TP to Alice and the sequence $S_{2}^{\prime }$ sent by Alice to TP. However, the attack methods that Bob may use cannot work, which has been analyzed in the previous part.

#### 5.2.2. The Attack from TP

The impact of a semi-honest third-party TP on the QPC protocol cannot be disregarded, as he/she communicates directly with the participants. In our protocol, TP participated in the preparation of GHZ-like state particles and the measurement of encoded quantum particles. TP may infer the secret information being compared based on the final measurement results, but this approach is not feasible. On the one hand, the user’s secret information is encoded on GHZ-like particles through unitary operations, and TP cannot know the specific unitary operations selected by the users. On the other hand, the two binary bits corresponding to each unitary operation are not actual secret information but ciphertext formed after performing a hash function. As a participant in the protocol, TP may also engage in side-channel attacks, which analyze and obtain relevant information of secret information from the physical characteristics of the system, such as the power consumption and processing time during the preparation or encoding of quantum particles. However, the application of decoy photon technology in this protocol can effectively interfere with the execution of side-channel attacks, while hash operations also avoid the leakage of true secret information. In addition, TP may adopt attack methods similar to Eve, but such attacks can be effectively resisted by our protocol. Therefore, the proposed protocol can resist an attack from TP.

## 6. Efficiency Analysis and Discussion

In this section, the quantum efficiency of our protocol is analyzed in detail. The quantum efficiency of a QPC protocol can be evaluated by comparing the number of classical bits to the number of quantum particles used in the comparison. It is well known that the efficiency of a QPC protocol can be expressed with the following equation [23]:

(24) $$\eta_{e}=\frac{\eta_{c}}{\eta_{t}}$$

where $\eta_{e}^{\;}$ denotes the QPC protocol’s efficiency, $\eta_{c}^{\;}$ denotes the number of compared classical bits in each comparison, and $\eta_{t}^{\;}$ denotes the number of generated particles in each comparison.

In the proposed protocol, we generated *n* three-particle GHZ-like states to compare *L* classical bits of secret data. After performing the hash operation, the length of classical bits transforms into *K*, which is given the value of 2*n*. The total number of quantum particles is 3*n*. Therefore, the quantum efficiency is 2n/3n=66%. The comparison of this protocol with other previously proposed QPC protocols is shown in Table 8.

## 7. Conclusions

A secure and efficient QPC protocol using GHZ-like states is proposed in this paper. Two quantum users, Alice and Bob, can compare the equality of their secret information with the help of a third-party TP. Compared with most previous QPC protocols, the proposed protocol is more efficient. And in the proposed protocol, the GHZ-like state is used to disseminate quantum information. The unitary operations are used to encode the GHZ-like state particles according to secret information encrypted with a shared hash function. The main feature of the protocol is that users can complete the comparison without sharing a quantum key and communicating, which makes the protocol greatly improved in terms of efficiency while ensuring security, and the protocol makes good use of quantum resources.

## Figures and Tables

**Table 1:** The operation outcome of the Pauli gates and Identity gate.

Qubit	X Gate	Y Gate	Z Gate	I Gate
|0〉	|1〉	−|1〉	|0〉	|0〉
|1〉	|0〉	|0〉	−|1〉	|1〉

**Table 2:** Summary table of $m_{i}$ and their corresponding unitary operations.

$m_{i}$	00	01	10	11
Unitary operation	*I*	*X*	*Y*	*Z*

**Table 3:** Summary table of equal results.

Measurement Method	Result 1	Result 2
$|0,1\rangle$ basis	$|0\rangle$	$|1\rangle$
Bell basis	$|\Phi^{+}\rangle$	$|\Psi^{+}\rangle$

**Table 4:** Summary table of all measurement results.

Alice	Bob	${|GHZ\rangle }_{1}{{}^{\prime }}_{①}$	Result	${|GHZ\rangle }_{1}{{}^{\prime }}_{②}$
I	I	$\left(|000\rangle +|011\rangle +|101\rangle +|110\rangle \right)/2$	Yes	$\left(\left|0\rangle \right|\Phi^{+}\rangle +\left|1\rangle \right|\Psi^{+}\rangle \right)/\sqrt{2}$
I	X	$\left(|001\rangle +|010\rangle +|100\rangle +|111\rangle \right)/2$	No	$\left(\left|0\rangle \right|\Psi^{+}\rangle +\left|1\rangle \right|\Phi^{+}\rangle \right)/\sqrt{2}$
I	Y	$\left(-|001\rangle +|010\rangle +|100\rangle -|111\rangle \right)/2$	No	$\left(\left|0\rangle \right|\Psi^{-}\rangle +\left|1\rangle \right|\Phi^{-}\rangle \right)/\sqrt{2}$
I	Z	$\left(|000\rangle -|011\rangle -|101\rangle +|110\rangle \right)/2$	No	$\left(\left|0\rangle \right|\Phi^{-}\rangle +\left|1\rangle \right|\Psi^{-}\rangle \right)/\sqrt{2}$
X	I	$\left(|010\rangle +|001\rangle +|111\rangle +|100\rangle \right)/2$	No	$\left(\left|0\rangle \right|\Psi^{+}\rangle +\left|1\rangle \right|\Phi^{+}\rangle \right)/\sqrt{2}$
X	X	$\left(|011\rangle +|000\rangle +|110\rangle +|101\rangle \right)/2$	Yes	$\left(\left|0\rangle \right|\Phi^{+}\rangle +\left|1\rangle \right|\Psi^{+}\rangle \right)/\sqrt{2}$
X	Y	$\left(-|011\rangle +|000\rangle +|110\rangle -|101\rangle \right)/2$	No	$\left(\left|0\rangle \right|\Phi^{-}\rangle +\left|1\rangle \right|\Psi^{-}\rangle \right)/\sqrt{2}$
X	Z	$\left(|010\rangle -|001\rangle -|111\rangle +|100\rangle \right)/2$	No	$\left(\left|0\rangle \right|\Psi^{-}\rangle +\left|1\rangle \right|\Phi^{-}\rangle \right)/\sqrt{2}$
Y	I	$\left(-|010\rangle +|001\rangle -|111\rangle +|100\rangle \right)/2$	No	$\left(\left|0\rangle \right|\Psi^{-}\rangle +\left|1\rangle \right|\Phi^{-}\rangle \right)/\sqrt{2}$
Y	X	$\left(-|011\rangle +|000\rangle -|110\rangle +|101\rangle \right)/2$	No	$\left(\left|0\rangle \right|\Phi^{-}\rangle +\left|1\rangle \right|\Psi^{-}\rangle \right)/\sqrt{2}$
Y	Y	$\left(|011\rangle +|000\rangle -|110\rangle -|101\rangle \right)/2$	Yes	$\left(\left|0\rangle \right|\Phi^{+}\rangle +\left|1\rangle \right|\Psi^{+}\rangle \right)/\sqrt{2}$
Y	Z	$\left(-|010\rangle -|001\rangle +|111\rangle +|100\rangle \right)/2$	No	$\left(\left|0\rangle \right|\Psi^{+}\rangle +\left|1\rangle \right|\Phi^{+}\rangle \right)/\sqrt{2}$
Z	I	$\left(|000\rangle -|011\rangle +|101\rangle -|110\rangle \right)/2$	No	$\left(\left|0\rangle \right|\Phi^{-}\rangle +\left|1\rangle \right|\Psi^{-}\rangle \right)/\sqrt{2}$
Z	X	$\left(|001\rangle -|010\rangle +|100\rangle -|111\rangle \right)/2$	No	$\left(\left|0\rangle \right|\Phi^{-}\rangle +\left|1\rangle \right|\Psi^{-}\rangle \right)/\sqrt{2}$
Z	Y	$\left(-|001\rangle -|010\rangle +|100\rangle +|111\rangle \right)/2$	No	$\left(\left|0\rangle \right|\Psi^{+}\rangle +\left|1\rangle \right|\Phi^{+}\rangle \right)/\sqrt{2}$
Z	Z	$\left(|000\rangle +|011\rangle -|101\rangle -|110\rangle \right)/2$	Yes	$\left(\left|0\rangle \right|\Phi^{+}\rangle +\left|1\rangle \right|\Psi^{+}\rangle \right)/\sqrt{2}$

where ${|GHZ\rangle }_{1}{{}^{\prime }}_{①}$ and ${|GHZ\rangle }_{1}{{}^{\prime }}_{②}$ denote the two expressions of ${{|GHZ\rangle }_{1}}^{\prime }$ performed unitary operations.

**Table 5:** The comparison process of equal secret information.

	Alice	Bob	TP
Secret information	X=101100011001	Y=101100011001	
After hash function H	$X^{\prime }=10110001$	$Y^{\prime }=10110001$	
Unitary operations	$\left\{Y,Z,I,X\right\}$	$\left\{Y,Z,I,X\right\}$	
$S_{2}^{\;}$ $\;\mathrm{and}\;S_{3}^{\;}$	$S_{2}^{\;}=\left\{|0\rangle ,|1\rangle ,|0\rangle ,|1\rangle \right\}$	$S_{3}^{\;}=\left\{|0\rangle ,|1\rangle ,|1\rangle ,|0\rangle \right\}$	$S_{1}^{\;}=\left\{|0\rangle ,|0\rangle ,|1\rangle ,|1\rangle \right\}$
$S_{2}^{\prime }$ $\;\mathrm{and}\;S_{3}^{\prime }$	$S_{2}^{\prime }=\left\{-|1\rangle ,-|1\rangle ,|0\rangle ,|0\rangle \right\}$	$S_{3}^{\prime }=\left\{-|1\rangle ,-|1\rangle ,|1\rangle ,|1\rangle \right\}$	
Combined pairs	$\left\{Q_{1},Q_{2},Q_{3},Q_{4}\right\}=\left\{|11\rangle ,|11\rangle ,|01\rangle ,|01\rangle \right\}$		
Measurement results	{$|\Phi^{+}\rangle$$,|\Phi^{+}\rangle$$,|\Psi^{+}\rangle$$,|\Psi^{+}\rangle$}		$\{|0\rangle ,|0\rangle ,|1\rangle ,|1\rangle$
Equality of secret	Yes		

**Table 6:** The comparison process of unequal secret information.

	Alice	Bob	TP
Secret information	X=101100011011	Y=101100011001	
After hash function H	$X^{\prime }=10110011$	$Y^{\prime }=10110001$	
Unitary operations	$\left\{Y,Z,I,Z\right\}$	$\left\{Y,Z,I,X\right\}$	
$S_{2}^{\;}$ $\;\mathrm{and}\;S_{3}^{\;}$	$S_{2}^{\;}=\left\{|0\rangle ,|1\rangle ,|0\rangle ,|1\rangle \right\}$	$S_{3}^{\;}=\left\{|0\rangle ,|1\rangle ,|1\rangle ,|0\rangle \right\}$	$S_{1}^{\;}=\left\{|0\rangle ,|0\rangle ,|1\rangle ,|1\rangle \right\}$
$S_{2}^{\prime }$ $\;\mathrm{and}\;S_{3}^{\prime }$	$S_{2}^{\prime }=\left\{-|1\rangle ,-|1\rangle ,|0\rangle ,-|1\rangle \right\}$	$S_{3}^{\prime }=\left\{-|1\rangle ,-|1\rangle ,|1\rangle ,|1\rangle \right\}$	
Combined pairs	$\left\{Q_{1},Q_{2},Q_{3},Q_{4}\right\}=\left\{|11\rangle ,|11\rangle ,|01\rangle ,-|11\rangle \right\}$		
Measurement results	{$|\Phi^{+}\rangle |,,\Phi^{+}\rangle ,|\Psi^{+}\rangle ,|\Phi^{-}\rangle$}		$\{|0\rangle ,|0\rangle ,|1\rangle ,|1\rangle$
Equality of secret	No		

**Table 7:** The example of the process that Eve eavesdrops (the decoy photon is $|+\rangle$).

The state of the decoy photon	$|+\rangle$			
The measurement basis Eve chooses	$|0,1\rangle$ basis			
The measurement result	$|0\rangle$		$|1\rangle$	
The measurement basis the receivers choose	X basis			
The measurement result	$|+\rangle$	$|-\rangle$	$|+\rangle$	$|-\rangle$
Is the result correct or not	Yes	No	Yes	No

**Table 8:** The comparison between our protocol and some previous protocols.

	Ref. [17]	Ref. [23]	Ref. [24]	Ref. [25]	Our Protocol
Quantum resource	eight-qubit entangled states	hyper-entangled GHZ states	five-qubit entangled states	four-qubit Cluster state and X-type state	three-particle GHZ-like state
QKD method	Yes	Yes	Yes	Yes	No
Decoy photon	Yes	Yes	Yes	Yes	Yes
Unitary operation	No	No	No	No	Yes
Entanglement swapping	No	Yes	No	No	No
Quantum efficiency	25%	66%	40%	50%	66%

## Data Availability

No new data were created or analyzed in this study. Data sharing is not applicable to this article.
